# The Hunt for the Putative Epoxyeicosatrienoic Acid Receptor

**DOI:** 10.1021/acschembio.5c00047

**Published:** 2025-03-24

**Authors:** William R. Arnold, Sona Jain, Vidya Sinha, Aditi Das

**Affiliations:** † Stanford Cryo-EM Center, Stanford University School of Medicine, Palo Alto, California 94305, United States; ‡ Departamento de Morfologia, Universidade Federal de Sergipe, São Cristóvão 49100-000, Sergipe, Brazil; § The Center for Advanced Studies in Science, Math and Technology at Wheeler High School, Marietta, Georgia 30068, United States; ∥ School of Chemistry and Biochemistry, Georgia Institute of Technology (GaTech), Atlanta, Georgia 30332, United States

## Abstract

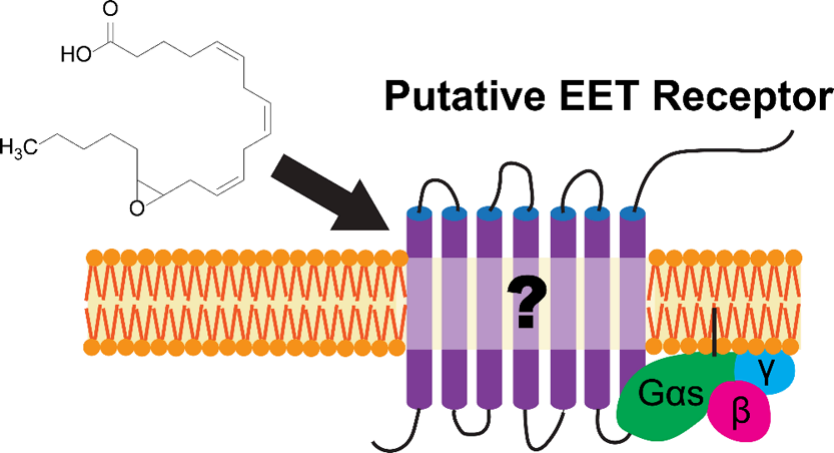

Epoxyeicosatrienoic acids, or EETs, are signaling molecules formed by the metabolism of arachidonic acid by cytochrome P450 enzymes. They are well-known for their anti-inflammatory effects, their ability to lower blood pressure, and benefits to cardiovascular outcomes. Despite the wealth of data demonstrating their physiological benefits, the putative high-affinity receptor that mediates these effects is yet to be identified. The recent report that the sphingosine-1-phosphate receptor 1 (S1PR1) is a high-affinity receptor for a related epoxy lipid prompted us to ask, “Why has the putative EET receptor not been discovered yet? What information about the discoveries of lipid epoxide receptors can help us identify the putative EET receptor?” In this review, we summarize the evidence supporting that the putative EET receptor exists. We then review the data showing EETs binding to other, low-affinity receptors and the discovery of receptors for similar lipid metabolites that can serve as a model for identifying the putative EET receptor. We hope this review will revitalize the search for this important receptor, which can facilitate the development of anti-inflammatory and cardiovascular therapeutics.

## 1 Introduction

**Figure 1:**
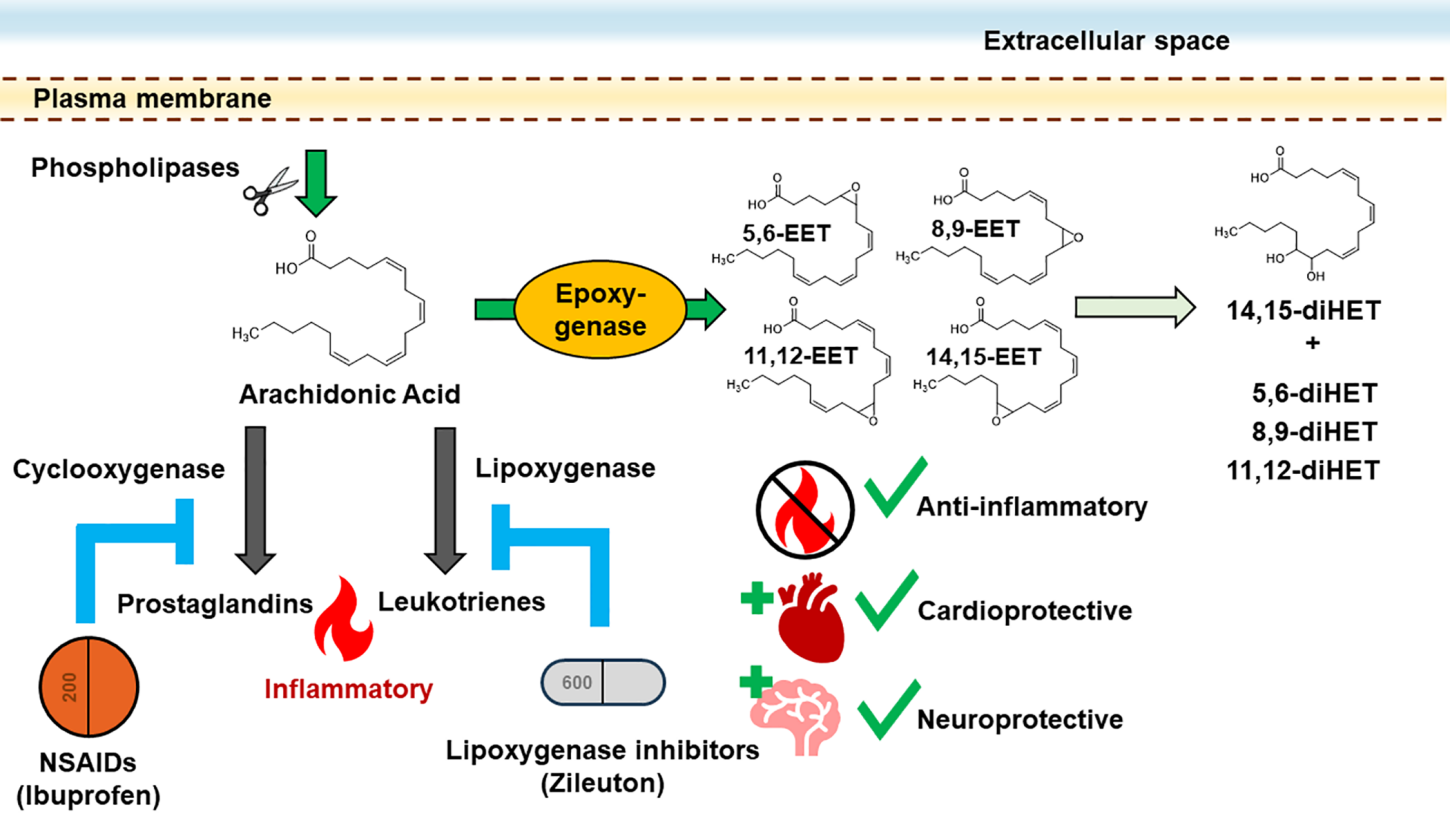
Arachidonic acid cascade and EETs. Phospholipases (phospholipases A2 and C) cleave arachidonic acid (AA) from the membrane during an inflammatory response. AA is then metabolized through three main pathways: the cyclooxygenase, lipoxygenase, and epoxygenase pathways. AA is transformed into epoxyeicosatrienoic acids (EETs) by the epoxygenase pathway. EETs are degraded into dihydroxyeicosatrienoic acids (diHETs) by soluble epoxide hydrolase (sEH).

Epoxyeicosatrienoic acids (EETs) are lipid-derived mediators involved in inflammation and cardiovascular health.^1^ Specifically, they are formed from the epoxidation of arachidonic acid (AA) by epoxygenases, which produce four regioisomers: 5,6-EET; 8,9-EET; 11,12-EET; and 14,15-EET (Figure 1).^2−5^ Known as the epoxygenase (EPOX) pathway, this branch of the arachidonic acid cascade is one of three branches of enzymes in eicosanoid synthesis. The other two branches are the cyclooxygenase (COX) and lipoxygenase (LOX) pathways (Figure 1). The COX and LOX enzymes convert AA into pro-inflammatory and often vasoconstrictive mediators known as prostaglandins (from COX) and leukotrienes (from LOX).^6^ NSAIDs and other drugs have been developed to inhibit these enzymes and reduce inflammation and the associated pain.

**Figure 2:**
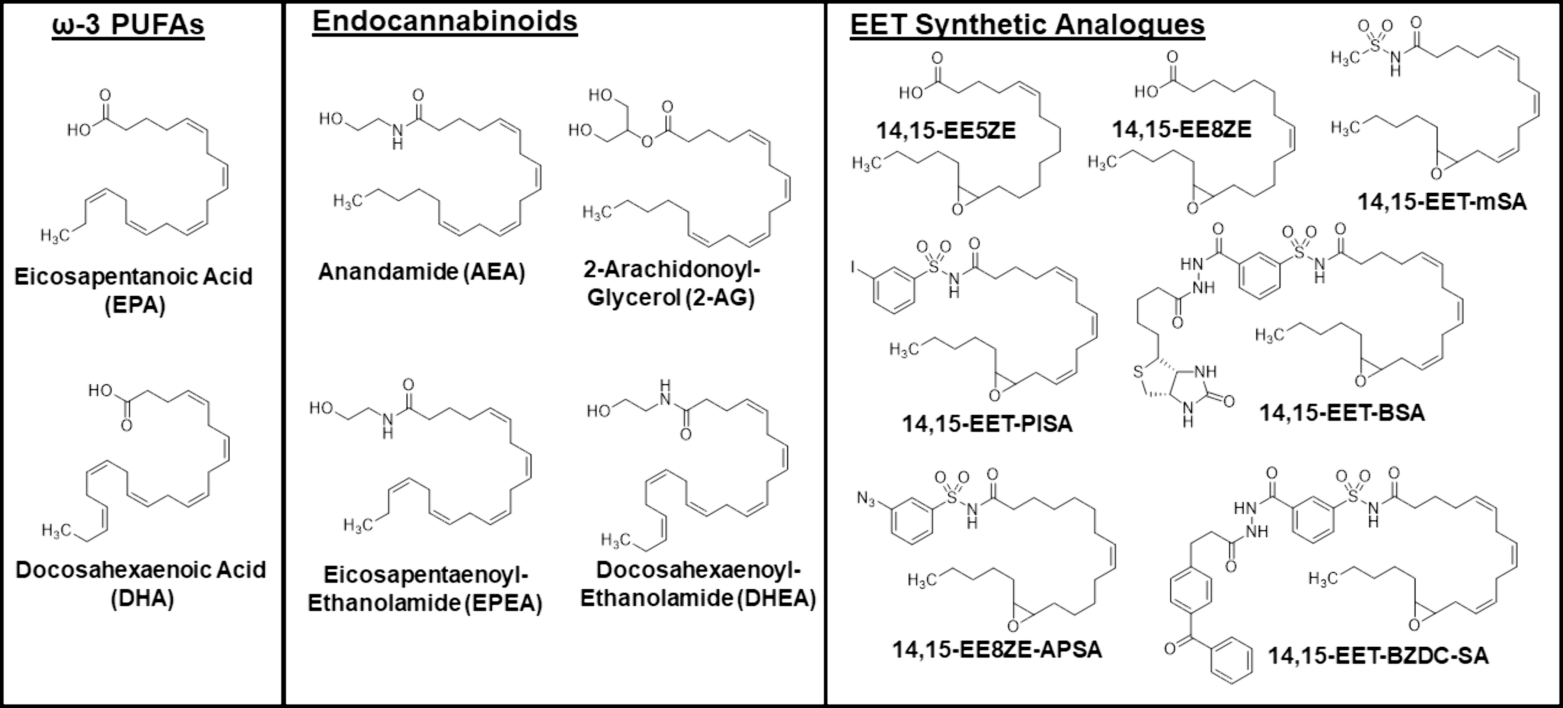
Structural analogues of AA and EETs. Other polyunsaturated fatty acids (PUFAs), such as ω-3 PUFAs, and endocannabinoids are metabolized by the EPOX pathway into epoxide metabolites that are structurally similar to EETs. Several synthetic analogues of EETs have been developed for receptor binding studies, and the structures of those mentioned in the text are shown.

The EPOX pathway differs from the COX and LOX pathways in that it produces EETs (and other epoxide mediators) that are generally anti-inflammatory, vasodilatory, mitobiogenic, and antiarrhythmic.^7−12^ The upregulation of EETs has been shown to help combat many cardiovascular-related diseases. For instance, EETs have been shown to reduce myocardial infarct size,^7^ reduce the cardiotoxic effects of doxorubicin,^13^ and attenuate alcohol-induced cardiac dysfunction.^14^ The main degradation pathway of EETs is through soluble epoxide hydrolase (sEH), which hydrolyzes EETs to dihydroxyeicosatrienoic acids (diHETs) (Figure 1). Many sEH inhibitors have been developed that increase levels of EETs *in vivo*,^15−17^ and these inhibitors have shown to lower blood pressure and improve kidney function,^18,19^ which shows the efficacy of targeting EET pathways in pharmacology. It is worth noting that EETs are also involved in angiogenesis and excessive levels of EETs have been shown to promote cancer in selected studies.^20,21^ The epoxygenases are specialized cytochromes P450 (CYPs), a superfamily of hemeproteins that oxidize lipophilic substrates. CYPs are best known for their role in phase I xenobiotic metabolism, inserting an oxygen atom into drugs to facilitate their elimination;^22^ however, epoxygenases (e.g., CYP2C8, CYP2C9, and CYP2J2) metabolize both drugs and polyunsaturated fatty acids (PUFAs) (Figure 2), with CYP2J2 having the highest expression in cardio(neuro)vascular tissues.^23−32^ It is worth noting that these CYPs perform other mono-oxygenation reactions such as hydroxylation, but for the purposes of this review we will focus on their epoxygenase activity.

Epoxides derived from many PUFA metabolites elicit similar effects to EETs, especially the ω-6-derived linoleic acid epoxides.^33^ Epoxides of ω-3 PUFAs have been shown to be more potent anti-inflammatory mediators^34,35^ and reduce cardiovascular disease.^36^ Thus, the benefit of ω-3 PUFAs has been partially attributed to their conversion to epoxide metabolites. Another important class of lipids, the endocannabinoids, are derived from PUFAs and are functionalized at the carboxylic headgroup with ethanolamine and glycerol moieties (Figure 2). They act as retrograde inhibitory neurotransmitters and as mediators of inflammation and vascular function, especially within the cerebral and nervous systems.^37−40^ Recently, epoxide metabolites of endocannabinoids have been discovered,^41−44^ which have demonstrated anti-inflammatory effects.^42^ However, unlike EETs, ω-3 and ω-3-endocannabinoid epoxides reduce tumorigenesis by attenuating proliferation.^45,46^

Despite the expanse of data supporting the benefits and functions of EETs and related epoxides, key details about the receptors that directly mediate these effects are currently lacking. Although EETs bind many different receptors, including ion channels and transcription factors, to mediate their functions,^11^ EETs are also postulated to bind to an unidentified surface G-protein coupled receptor (GPCR) termed the putative EET receptor. This receptor is postulated to be responsible for many of the vasorelaxation and anti-inflammatory effects of EETs. Therefore, its identity is crucial to understanding applications of the EET pathway to cardiovascular and inflammatory therapeutics. Herein, we review the data demonstrating the existence of the putative EET receptor. We will also review other low-affinity receptors for EETs and similar molecules. Finally, we provide examples involving similar lipid receptors of other eicosanoids that serve as models for discovering EET receptors.

## 2 The Putative Endothelial EET Receptor

Here we examine the evidence for the presence of a vascular endothelial EET receptor and its key characteristics. Given that the evidence points toward the receptor being a G-protein-coupled receptor (GPCR), we will start with a concise introduction to GPCR structure, function, and signaling mechanisms. For an in-depth exploration of GPCR physiology, we refer the reader to the many focused reviews on GPCRs.^47−49^

### 2.1 Introduction to GPCRs

GPCRs represent the largest superfamily of proteins in the human genome, numbering 826 members. GPCRs are expressed on the plasma membrane and respond to a variety of stimuli, transducing intracellular signals that spearhead a multitude of physiological processes. As a result, this superfamily is an important pharmacological target, with nearly 20% of all GPCRs being confirmed drug targets and over 500 of the drugs on the market targeting these receptors.^48^ However, there remain over 100 GPCRs that are considered orphans, meaning that their native ligand or mode of activation have not been determined and their function may not be fully understood.^47^

Although GPCRs are classified into different classes based on structural and ligand archetypes, all GPCRs have a characteristic seven transmembrane helical domain and varying sizes of extracellular and intracellular domains. By far, Class A (“rhodopsin-like family”) contains a majority of all GPCRs and will be focal point of this review. Class A GPCRs are activated by a multitude of peptides, neurotransmitters, hormones, sensory stimuli (e.g., light), and lipids that generally target the transmembrane domain. About half of the Class A members are olfactory receptors and are responsible for detecting the various scent and taste molecules we encounter to sense our chemical environment, including the food we eat. As such, their expression is primarily limited to olfactory cells in the mouth and nose. Therefore, when studying the actions of endogenous stimuli, especially in the context of deorphanizing a receptor, these olfactory receptors are largely ignored, as it is unlikely that these receptors contribute to other physiological processes.^47−49^

### 2.2 G Proteins and Arrestins

GPCRs are so named due to their interactions with a heterotrimeric G protein complex composed of Gα, Gβ, and Gγ units. These G proteins are expressed on the inside of the cell and interact with GPCRs at their cytosolic face. The Gα unit is a guanine nucleotide exchange factor that is inactive when bound with GDP. The inactive (GDP) form of Gα forms a complete heterotrimer with the Gβ and Gγ units, and the heterotrimer binds to GPCRs in their resting state. GPCR activation induces a conformational change allowing the Gα unit to disengage with the Gβ-Gγ dimer and for the Gα unit to exchange GDP for GTP. The G proteins, in turn, disengage with the GPCR and initiate the intracellular signaling pathways. The termination of GPCR activation (and signaling) is initiated by C-terminal phosphorylation of the activated GPCR by GPCR kinases (GRKs), which increases binding affinity to arrestins (e.g., β-arrestin). The binding of arrestins physically obstructs GPCR binding to G proteins and often leads to cellular uptake of the GPCR/arrestin complex.^47−49^

There are several Gα units that are classified in 4 broad categories based on the signaling pathway they activate. The Gα_S_ unit (G_S_) stimulates adenylyl cyclase activity to produce cyclic adenosine monophosphate (cAMP). cAMP, in turn, activates protein kinase A (PKA), leading to the phosphorylation of downstream proteins. The Gα_i/o_ units (G_i/o_) are the antithesis of the G_S_ pathway. Instead of activating, they inhibit PKA and quiet cAMP signaling. The Gα_q/11_ units activate phospholipase C (PLC), which in turn produces diacylglycerol (DAG) and inositol triphosphate (IP_3_). DAG activates protein kinase C (PKC) while IP_3_ stimulates Ca^2+^ release and thus Ca^2+^-dependent protein activation. Lastly, the Gα_12/13_ units (G_12/13_) are involved in cytoskeletal rearrangement during cellular movement and migration.^47−49^

This simple model of GPCR signaling pathways, however, is continually being challenged. For instance, the Gβ-Gγ dimer that breaks off from G_S_ can stimulate PLC activation and the Gβ-Gγ dimer that breaks off from G_q_ can stimulate adenylyl cyclase. Adding further complexity, the same GPCR can bind with multiple different types of G protein complexes. Exploring the complexity of GPCR signaling is beyond the scope of this review, but it is worth keeping in mind when thinking about unidentified GPCR mediators.^49^

The GRKs and arrestins are considered the “deactivation” pathways of GPCR signaling, as they help to return GPCRs to an inactivated state. However, GRKs and arrestins initiate their own signaling pathways that expand the functional outcomes of the GPCR pathway.^50−52^ β-Arrestins contain binding domains that associate with clathrin endocytic machinery and other proteins mediating cellular uptake. β-Arrestins also stimulate pathways that are complementary to G-protein pathways and often antagonize G-protein signaling. These complementary pathways include PI3K, ERK1/2, and Akt. The growing examples of β-arrestin signaling is gaining pharmaceutical interest, as tapping into this phenomenon can aid in drug design, either by avoiding or promoting β-arrestin signaling.^51,52^ Such is possible because different ligands can alter which pathways a particular GPCR interacts with, a process known as “ligand bias” that is discussed below.

### 2.3 Ligand Bias

The “one ligand, one receptor” paradigm is continually being challenged, especially in the GPCR field. This paradigm states that a receptor binds to only one ligand that induces a specific conformation mediating activity. Not only are there many examples of unique GPCRs binding many ligands (including nonolfactory GPCRs) but different ligands can induce a spectrum of conformations that vary receptor function. For example, the binding of Ligand A to a GPCR can induce a G_i_ phenotype, but the binding of Ligand B to the same GPCR can induce a G_s_ phenotype, as observed with cannabinoid receptors.^53^ This process (known as “ligand bias”) should be considered when determining the functions of uncharacterized ligands and GPCRs.^54−56^ Crucially, this means that a nonbiased investigation of GPCR may be necessary to identify the receptor of a new ligand. We cannot rule out that the receptor for the ligand is already known to bind another ligand; further, that receptor may not canonically mediate the same responses as the putative ligand.

### 2.4 Evidence that EET Interacts with a Cell-Surface Membrane Receptor (GPCR)

The earliest reports on the presence of a cell-surface EET receptor were published by Wong and colleagues in the 1990s.^57−59^ They identified binding sites for [^3^H]-14,15-EET in U-937 monocytes with high affinity (K_d_ of 13.84 ± 2.58 nM) and a *B*_max_ of 3.54 ± 0.28 pmol/10^6^ cells. Unlabeled 14(R),15(S)-EET or 14(S),15(R)-EET could easily dissociate cell-bound [^3^H]-14,15-EET in a time-dependent manner, with 14(R),15(S)-EET being more effective than the 14(S),15(R)-isomer. This enantiomer selectivity points to a specific binding site. The binding of [^3^H]-14,15-EET to U-937 cells was also protease-sensitive, demonstrating that the radiolabeled EET bound to a protein receptor and not nonspecifically to the membrane. Cholera and pertussis toxins, known to increase intracellular cAMP levels (as well as compete with Gα subunits for binding), were shown to decrease [^3^H]-14,15-EET receptor binding by 45.87 ± 1.97% and 38.77 ± 1.83%, respectively, without changing the binding affinity of [^3^H]-14,15-EET. Similarly, the addition of dibutyryl cAMP reduced the binding of [^3^H]-14,15-EET to the cell surface, which could be reversed by a PKA inhibitor. These findings indicate the involvement of a G_S_-coupled GPCR. A specific binding site for 14,15-EET, and cAMP- and PKA-related mechanisms was also reported in guinea pig monocytes (GPM) in one of their later studies.^59^ Wong and colleagues thus demonstrated that the binding of 14,15-EET to a surface receptor in U937/GPM cells results in an increase in intracellular cAMP, which activates a PKA-dependent signaling pathway in these cells. However, no receptor protein was cloned or purified.

Later, several radiolabeled 14,15-EET agonists and antagonists were developed that confirmed the binding of 14,15-EET to membranes of U937 cell lines. Based on their previous work with 14,15-EET-mSA (methylsulfonamide,^60^), Campbell and colleagues developed three 14,15-EET agonists: 14,15-EET-PISA, 14,15-EET-BSA, 14,15-EET-BZDC-SA (Figure 2).^61^ These 14,15-EET-mSA derivatives were produced by replacing the carboxylate headgroup with an N-acylsulphonamide group, which has a similar p*K*_a_ as a carboxylate but prevents β-oxidation and phospholipid esterification. All three analogues were as effective as 14,15-EET in mediating coronary artery relaxation. Vasorelaxation by these analogues was blocked by a calcium-sensitive potassium channel (K_ca_) inhibitor, an EET antagonist, and extracellular K^+^. Later, Campbell and colleagues developed an iodinated benzyl group that was attached to the sulphonamide group of 14,15-EET-PISA to produce the radiolabeled analogue 14,15-EET-P^125^ISA.^61^ Although they showed that 14,15-EET-P^125^ ISA could metabolize to 14,15-DEET-P^125^ISA (the diHET congener) by sEH, it was still metabolically stable enough for performing radioligand binding experiments to the membrane fraction of U937 cells that contains the receptor. Binding was concentration-dependent and reached a maximum plateau at 100 nM with a predicted *K*_d_ and *B*_max_ of 148.3 ± 36.4 nM and 3.3 ± 0.5 pmol/mg protein, respectively. To confirm that this binding was specific, similar experiments were also performed using 14,15-DEET-P^125^ISA, which showed very low binding to U937 cell membranes. This confirmed that 14,15-EET-P^125^ISA binding was specific, and that a 14,15-epoxide group was required for 14,15-EET-P^125^ISA to bind to U937 membranes.

Campbell and co-workers further supported the presence of an EET receptor by developing radiolabeled analogues of the EET agonist 14,15-EE8ZE (20-I^125^-14,15-EE8ZE)^62^ and the EET antagonist 14,15-EEZE (20-I^125^-14,15-EE5ZE) (Figure 2).^63^ Importantly, these radiolabeled analogues were as efficacious as their unlabeled counterparts. 20-I^125^-14,15-EE5ZE bound to U937 cell membranes in a concentration-dependent manner and was saturable. In competition binding experiments, EETs (8,9-EET; 11,12- EET; and 14,15-EET), but not EET derivates (HETEs or DiHETs), competed for 20-I^125^-14,15-EE5ZE. 11,12-EET displaced the radioligand by 50% in less than 10 min and completely displaced it within 30 min.^63^ The rate of displacement of 20-I^125^-14,15-EE5ZE by 11,12-EET^63^ was slower than the displacement of the synthetic agonist 20-I^125^-14,15-EE8ZE by 11,12-EET,^62^ suggesting that 20-I^125^-14,15-EE5ZE has a higher affinity. These data also indicated that the binding of 20-I^125^-14,15-EE5ZE is reversible. Since GTPγS blocked 20-I^125^-14,15-EE8ZE signaling,^62^ it was concluded that 14,15-EE8ZE (and thus EETs) bind to a GPCR.

**Figure 3:**
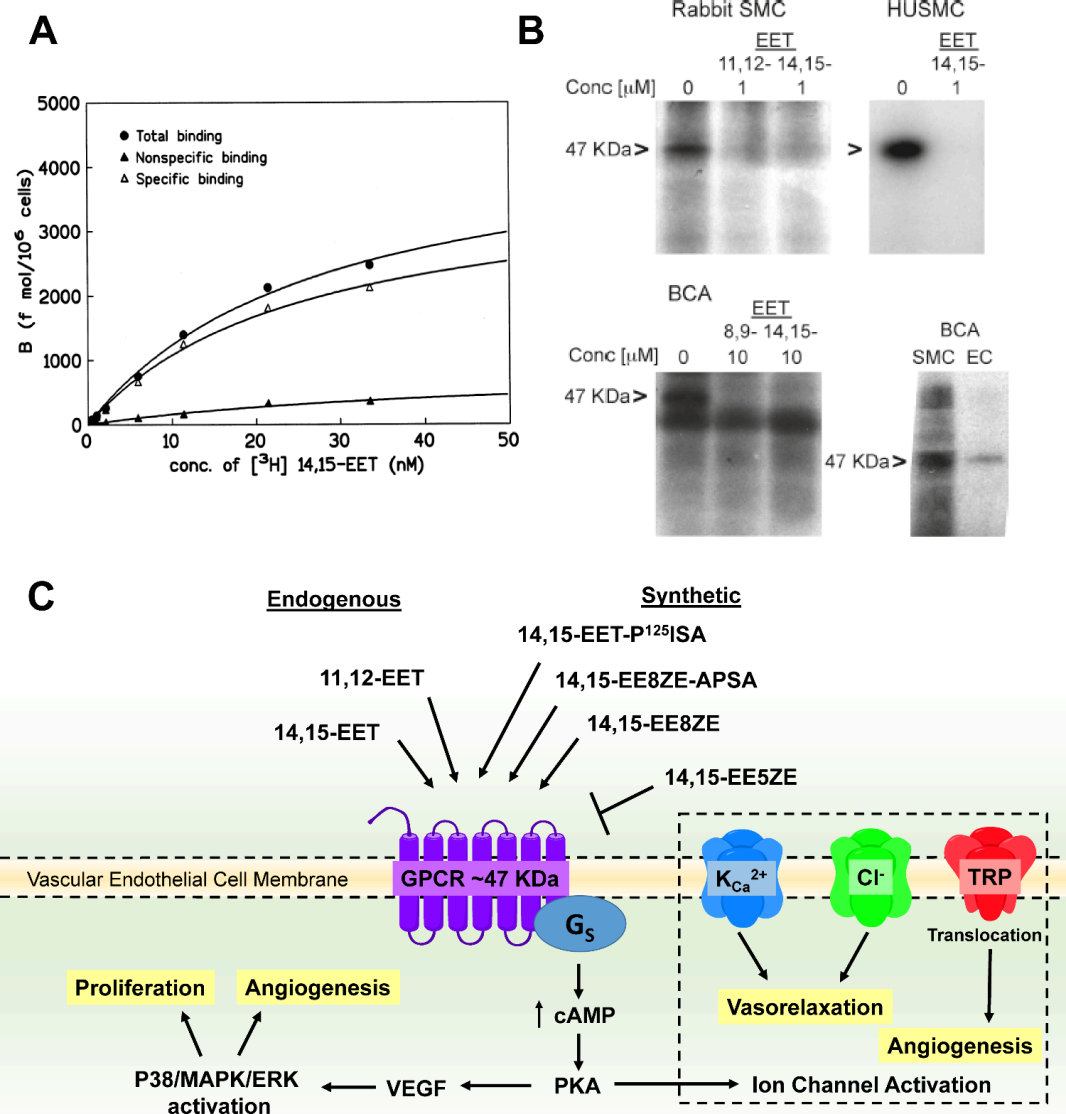
Putative EET receptor. A. Radiolabeled 14,15-EET binding to U937 cell membrane fractions. Data shows binding is saturable and specific. Data is reprinted with permission from Wong et al.^56^ Copyright 1997, Elsevier. B. 20-I^125^-14,15-EE8ZE-APSA binding to rabbit smooth muscle cells (SMC), human umbilical smooth muscle cells (HUSMC), Bovine coronary artery (BCA) SMC and endothelial cells (EC). A prominent 47 kDa band is observed in all these preparations. Reprinted (adapted) with permission from Chen et al.^64^ Copyright 2011, American Chemical Society. C. Graphical summary of the characteristics of the EET receptor detailed in Section 2. Structures of agonists and antagonists can be found in Figure 2.

A major advance in the search for the putative EET receptor came from the synthesis of a radiolabeled and photoactive analogue, named 20-I^125^-14,15-EE8ZE-APSA (Figure 2).^64^ This analogue contains an additional phenylsulfonamide group with a photoactive azide, allowing for covalent labeling of the EET receptor/binding site via UV-induced cross-linking. This analogue was used in electrophoresis assays of cell membrane fractions that visualized a radioactive protein band 47 kDa in size. The labeling of the 47-KDa band was inhibited by 11,12-EET and 14,15-EET in mixed cell membranes and by 14,15-EET when only plasma membranes were used. Concentration-dependent competition (10^–10^ to 10^–5^ M) for photolabeling was performed with EETs (8,9-EET, 11,12-EET, and 14,15-EET); 14,15-EE5ZE; and a structurally similar-but-biologically inactive analogue (14,15-thiirane). As expected, 14,15-EET; 11,12-EET; 8,9-EET; and 14,15-EE5ZE inhibited the photolabeling while 14,15-thiirane did not. It was thus concluded that the 47-KDa band represents a high-affinity receptor or receptor domain for 11,12-EET and 14,15-EET but has lower affinity for 8,9-EET and 14,15-EE5ZE. Photolabeling experiments were also performed in membranes from bovine coronary artery, vascular smooth muscle, endothelial cells, canine heart, and rat kidneys, and the 47-KDa band was present for all preparations (Figure 3B). Based on its 47-kDa size, a GPCR was postulated.^64^

20-I^125^-14,15-EE8ZE-APSA was also used in a GPCR binding screen of 79 orphan GPCRs that (1) bind lipids, (2) are phylogenetically related to lipid binding receptors, or (3) are expressed in EET-responding tissues. HEK293T cells were transfected with one of each 79 N-terminal FLAG-tagged GPCRs and the cell lysates were incubated with 20-I^125^-14,15-EE8ZE-APSA. However, these photolabeling experiments could not detect a receptor in HEK293T cells overexpressing these GPCRs. It was concluded that none of the 79 GPCRs tested represent the high-affinity receptor for 14,15-EET.^64^ Similar results were also published by another group, where 105 GPCRs of interest were expressed in *Xenopus* oocytes and subsequently were screened for the increase of cAMP-dependent chloride current after treatment with 14,15 EET.^65^ Furthermore, an ERK activation assay and a β-arrestin recruitment assay were utilized as alternative methods, where 241 GPCRs, including 73 orphan GPCRs, were screened.^65^ Although no high-affinity receptors could be identified from these screens, the authors still maintain the presence of high affinity receptor for 14,15-EET based on the evidence of the reports.

### 2.5 Further Evidence of GPCRs: Interactions with Ion Channels

Further evidence for the existence of an EET GPCR comes from several observations of direct or indirect interactions of EETs with ion channels. In 1996, Campbell and co-workers^66^ first proposed that 11,12-EET served as an endothelium-derived hyperpolarizing factor (EDHF) that acted on bovine smooth muscle by the activation of K_Ca_ channels. However, the mechanism by which EETs dilate coronary arteries and hyperpolarize vascular smooth muscle remained unknown. In 1997, a study by the same group^67^ indicated that 11,12-EET activates the K_Ca_ channels by a G_S_-mediated mechanism. In the cell-attached patch mode, 11,12-EET caused a concentration dependent increase in the activity of K_Ca_ channel with concentrations as low as 1 nM. 12-hydroxeicosatetraenoic acid (12-HETE), a structural analogue of 11,12-EET, had no effect on the activity of the K_Ca_ channels, while 20-HETE decreased the activity of this K_Ca_ channel. These data indicate that the epoxide group is required for K_Ca_ channel activation. In the inside-out excised membrane patch mode, 11,12-EET had no effect on the activity of the K_Ca_ channel unless GTP (0.5 mM) was added to the bath solution, which suggests that the EET-evoked responses require a cytosolic component or a cellular signaling pathway that is absent in inside-out patches and which requires GTP. GDP-β-S (G-protein inhibitor) reduced the activity of the K_Ca_ channels by 16% and completely abolished the effect of 11,12-EET, further specifying that a GTP and a G-protein-linked mechanism was involved. The addition of anti-G_S_ antibody to the cytosolic side was able to block currents evoked by 11,12-EET applied to the extracellular side, while anti-Gβγ and anti-G_i_ had no effects. Western blot of the smooth muscle homogenate with anti-G_S_ antibody gave two protein bands of approximately 52 and 45 kDa, which were similar to the results obtained with the G_S_ standard. These findings support the presence of a surface receptor in bovine coronary arteries for 11,12-EET that activates a G_S_-coupled GPCR, which subsequently activates the K_Ca_ channel. Since, no increase in intracellular cAMP was observed in response to 11,12 EET^66^ and ATP was not required to restore the effect of 11,12-EET on the channel,^67^ it was proposed that there is a cAMP/PKA-independent mechanism of action of G_S_ on the K_Ca_ channel. A PKA-independent pathway was also proposed for the inhibitory effect of EET on Cl^–^ channels in rat mesenteric arterial smooth muscle.^68^ Membrane-delimited action of G_S_ for regulating ion channels has been reported in many studies,^68−70^ so this report by Li and Campbell^67^ also supports the presence of a membrane receptor for EET, most probably a GPCR associated with G_S_.

Bioactive hormones and secondary messengers have been reported to regulate ADP-ribosylation of cellular proteins, especially G proteins, thereby modulating intracellular signaling. EETs, particularly 14,15-EET, was shown to stimulate ADP-ribosylation in the liver cells.^71^ Later, Li and Campbell^72^ showed in one of their studies that ADP ribosylation of G_S_ was related to K_Ca_ channel activation in response to 11,12-EET. Autoradiography of endogenous ADP-ribosylation in bovine coronary artery smooth muscle cells gave 51-, 52-, 80-, and 124-kDa protein bands, and the density of the 52-kDa band was markedly increased in the presence of 11,12-EET and GTP. Cholera toxin also stimulated the ADP-ribosylation of 45-, 52-, and 80-kDa proteins. However, pretreatment of the homogenate with 11,12-EET significantly inhibited the ADP-ribosylation of these proteins by cholera toxin in a concentration-dependent manner, suggesting that the 11,12-EET-induced endogenous ADP-ribosylation may competitively inhibit cholera-toxin-induced ADP-ribosylation. Western blot using anti-G_S_ antibody immunoprecipitated a 52-kDa band, demonstrating that the 11,12-EET-induced 52-kDa band corresponded to G_S_. The stimulating effect of 11,12-EET on the K_Ca_ channel was blocked in the presence of mono ADP-ribosyltransferase inhibitors. In summary, this study demonstrated that 11,12-EET-induced activation of K_Ca_ is possibly carried out via ADP-ribosylation of the G_S_ subunit of GPCRs.^72^

Many EET-induced biological responses, such as cell proliferation, gap junctional communication, or transient receptor potential channel (TRP) translocation are dependent on the activation of protein kinase A (PKA), which suggests the existence of a G_S_- coupled receptor for EET. Also, production and activity of EET regioisomers and enantiomers have been shown to vary in different tissues and species. Ding and colleagues^73^ reported the enantiomer-specific response of 11,12-EET for the translocation and activation of TRP channel and angiogenesis in primary cultures of human endothelial cells. Racemic 11,12-EET and 11(R),12(S)-EET resulted in rapid translocation of a TRPC6-V5 fusion protein and stimulated angiogenesis, but 11(S),12(R)-EET did not. These effects on TRP channel and angiogenesis were sensitive to EET antagonists. TRP channel trafficking was also prevented by a PKA inhibitor. On the other hand, RNAi-mediated downregulation of Gα_s_ abolished responses to racemic 11,12 EET but failed to affect migration and tube formation in endothelial cells stimulated either by vascular endothelial cell growth factor (VEGF) or a PKA activator, suggesting that these signaling molecules function downstream of G_S_. Thus, this work confirms the presence of a G_S_-coupled membrane receptor for the PKA-dependent translocation and activation of TRPC6 channels and angiogenesis in human endothelial cells induced by 11(R),12(S)-EET.

### 2.6 Final Remarks on the Putative EET Receptor

The several reports of physiological responses elicited by low concentrations of EETs suggest the existence of high-affinity EET receptors.^11,31^ Furthermore, the ligand binding studies mentioned above implicate the presence of a cell surface, high-affinity receptor that is most probably a G_S_-coupled GPCR approximately 47 kDa in size. Despite this overwhelming evidence, no EET receptor has been molecularly cloned and identified despite two attempts at GPCR high-throughput screening. There could be many reasons why these attempts have failed. The *in vitro* assay systems and the cell lines used for receptor screening could be lacking some essential cytosolic component necessary for receptor identification that is present in cells that natively express this receptor. Another possibility that obfuscates the identity is that multiple GPCRs (or other receptors) could be acting in concert (as may be demonstrated by Section 3), and thus investigating one GPCR will not fully produce the observed phenotype seen from the native cells. There is also the possibility that the receptor has already been discovered, but that it mediates the functions of an already established ligand, as exemplified by the recent discovery of 17,18-EEQ binding to the sphingosine-1-phosphate receptor (see Section 4.1). Therefore, an unbiased approach that looks at several GPCRs (not just orphaned or lipid-binding) may be needed. Mass spectrometry analysis of the 47-KDa radiolabeled band^64^ is an example of such an approach.

It is difficult to speculate on a negative result, creating uncertainty as to why these screens failed to identify the receptor in question. To better understand how we may discover this putative receptor, we should also review what receptors are already known to bind EETs (Section 3) and other lipid epoxides (Section 4).

## 3 EET Binding to Known Membrane Receptors

Although the putative high-affinity GPCR of EETs is yet to be elucidated, many other EET receptors have been discovered. In this next section, we will review other identified receptors of EETs, which have either shown to have low affinities for EETs or do not fully meet the phenotypical criteria for the putative EET receptor. It is worth noting that several studies have shown that various receptors are involved with EET physiology. For example, the work of Li et al. shows that Gα_12/13_ is involved in zebra fish embryonic hematopoiesis.^74^ However, we have focused this review on studies that investigate direct binding to receptors at the biochemical level in order to give us insight into the putative EET receptor.

### 3.1 GPCRs

#### 3.1.1 GPR40

GPR40 is a fatty acid receptor that is expressed in pancreatic β cells, whereby it contributes to insulin regulation.^75^ GPR40 has also been shown to be expressed in several human vascular endothelial cells and was shown to mediate the mitogenic response to EETs in kidney epithelial cells.^76^ Campbell and colleagues later characterized GPR40 as a low-affinity vascular endothelial receptor of EETs.^77^ A fluorescent calcium-influx assay was used to determine G-protein activation in HEK-293 cells overexpressing GPR40. EETs, especially 11,12- and 14,15-EET (EC_50_ = 0.91 ± 0.08 and 0.58 ± 0.08 μM, respectively), promoted calcium influx in a concentration-dependent manner; untransfected cells did not respond to EETs. These results were mimicked by a GPR40 agonist, GW9508. Influx was absent when calcium was removed from the extracellular medium, demonstrating that the influx is due to extracellular calcium and not mitochondrial or intracellular stores. Neither diHETs nor thiirane derivatives of 11,12- and 14,15-EET were as potent as the EETs, and *cis-* or *trans-*EETs had similar effects. Antagonizing GPR40 with GW1100 or knocking down GPR40 with siRNA also reduced the effects of EETs. Next, the ability of EETs to inhibit radiolabeled TAK-875 (a GPR40 agonist) binding was assessed in the HEK-GPR40 cells. 11,12- and 14,15-EET were able to inhibit TAK-875 binding with *K*_i_ values of 2.7 and 6.4 μM, respectively. GPR120 is a related receptor to GPR40 and was also tested in the calcium assay; however, GPR120 activation was weaker compared GPR40.

The biological activity of GPR40 was then assessed in pancreatic cell lines INS-1 832/13, which natively express this receptor. Likewise, the EETs were able to promote Ca^2+^ influx in these cells, which was antagonized by GW1100. The ability of 11,12-EET to promote vasorelaxation through GPR40 was assessed in bovine coronary arteries. Although 11,12-EET could relax arteries preconstricted with the thromboxane mimic U46619, GW1100 could not antagonize this effect, demonstrating that the vasorelaxation by 11,12-EET is GPR40-independent. Finally, inflammatory markers were assayed in HUVEC cells and demonstrated that 11,12-EET-dependent phosphorylation of ERK, gap junction Cx43 expression, and COX-2 expression were antagonized with GW1100, suggesting a GPR40-mediated pathway was at least partially mediating these effects. However, since high amounts of EETs were needed and vasorelaxation was not mediated by GPR40, GPR40 was ruled out as being the putative EETs receptor.^77^

#### 3.1.2 Thromboxane Receptors

As EETs and prostaglandins are both derived from AA, it is not surprising that there has been reported crosstalk between EETs and prostanoid receptors. Prostanoid receptors are GPCRs and are classified into five types—DP, EP, FP, IP and TP—based on the sensitivities to five naturally occurring prostaglandins—PGD2, PGE2, PGF2, PGI2 and thromboxane A2 (TxA2), respectively.^78^ Behm and colleagues found that EETs inhibit TP receptors.^79^ After demonstrating that EET-induced vasorelaxation in aorta from knocked-out mice for either BK_Ca_ or transient receptor potential vanilloid 4 (see Section 3.2), Behm and colleagues found that vasoconstriction induced by the TP receptor agonist U-46619 was directly and competitively antagonized by 14,15-EET. 14,15-EET failed to inhibit phenylephrine-induced vasoconstriction, proving that it is specific to TP. 14,15-DHET was as effective as 14,15-EET, which is atypical since DHETs usually do not elicit similar responses as EETs. Using various vascular tissue from rat, mice, and guinea pigs, the authors demonstrated further that the vasorelaxation was not due to adrenergic or muscarinic receptors, and that among the prostanoid receptors, 14,15-EET was most effective against TP receptors, weaker against prostaglandin receptors, and showed no activity with leukotriene receptors. Ultimately, the inhibition of TP receptors contradicts the activation phenotype of the putative EET receptor, thus eliminating TP receptors as the putative receptor.

#### 3.1.3 Prostaglandin Receptors

EPs are subdivided into four types,^80^ of which EP2 and EP4 are G_S_-coupled GPCRs linked to vasodilation.^81^ In a study published in 2010,^82^ Yang and colleagues showed that the relaxant effect of EET in rat mesenteric arteries occurs at least partially via EP2 receptor stimulation. 14,15-EET induced concentration-dependent relaxation in rat mesenteric arteries, which was reduced by a selective G_S_ inhibitor, cAMP antagonist, and PKA inhibitor. Pretreatment of mesenteric arteries with an EP2 receptor antagonist also reduced the 14,15-EET relaxant effect by two-thirds, but not completely. PGE2, the typical EP2 receptor agonist, could mimic the effects of 14,15-EET. The binding of 14,15-EET to the cell membrane was also shown to be modified by an EP receptor antagonist and RNA silencing of EP2 receptors. Another report by Liu and Colleagues supported the hypothesis that EP2 receptor is involved in mediating the effects of 14,15-EET.^65^ As EP2 receptor antagonists could only attenuate, but not completely block, the relaxing effects of 14,15-EET, Yang and colleagues concluded that receptors other than EP2 are involved.^82^ In their subsequent study,^83^ they showed the involvement of the K_Ca_ channel in addition to EP2 receptor in EET-induced vasorelaxation. These effects still could not be totally abolished by the combined efforts of EP receptor antagonism and K_Ca_ channel blocking, indicating the involvement of other unknown mechanisms. Therefore, due to the lower apparent affinity of EETs toward EP2 and the fact that inhibition did not fully block their affects, it is doubtful that EP2 is the putative EET receptor.

### 3.2 Transient Receptor Potential (TRP) Channels

TRP channels are a major superfamily of (mostly) nonselective cation channels mediating several physiological activities. These channels are known for being prominently regulated by lipids, both directly and indirectly. Importantly, many of these channels are regulated by inflammatory lipids; thus, it is no surprise that many of these channels have been shown to be direct receptors of lipid epoxides. Here, we summarize the data demonstrating that lipid epoxides, especially EETs, target TRP channels.

#### 3.2.1 TRPV4

Transient receptor potential (TRP) vanilloid 4 (TRPV4) is expressed in several tissues, including endothelial, smooth muscle, skeletal, and neuronal tissues, and is activated by shear-stress and bioactive lipids.^84−86^ Importantly, TRPV4 activation leads to vasodilation that is implicated in cardiovascular health.^87^ The work of Watanabe and colleagues in 2003 showed that 5,6-EET derived from either AA or AEA is a direct agonist of TRPV4. Using HEK293 cells expressing TRPV4, they discovered that both AA and AEA produced a robust calcium influx comparable to the synthetic agonist, 4α-phorbol-12,13-didecanoate (4αPDD). Neither a nonhydrolyzable analogue of AEA (preventing conversion to AA) nor a nonmetabolizable analogue of AA produced TRPV4 responses; furthermore, applying AA or AEA to inside-out patches failed to produce a response. These data indicated that a metabolite of AA is responsible for mediating the effects. The CYP inhibitor miconazole abrogated TRPV4 activation by either AA or AEA and demonstrated that the metabolite was CYP-derived. They found that 5,6-EET had the most robust response; 8,9-EET had a minor response; and 11,12-EET, 14,15-EET, and 20-HETE had no effect. They further demonstrated that the activity of 5,6-EET on mouse aorta endothelial cells was blocked by ruthenium red (a TRP channel blocker) and is independent of large-conductance Ca^2+^-activated K^+^ channels, indicating the TRPV4 is the primary receptor responsible for 5,6-EET vasoactivity.

Later, the combined efforts of Vriens and colleagues^88^ and Fernandes and colleagues^89^ showed that the mechano/osmo-sensation of TRPV4 relies on 5,6-EET. As cell swelling leads to phospholipase A_2_ (PLA_2_) activation, Vriens et al. explored the possibility that TRPV4 activation during cell swelling is mediated through the PLA_2_ pathway. TRPV4 was transfected into HEK293 cells, whereby selected PLA_2_ inhibitors (that are not molecular analogues of AA) were able to block TRPV4-mediated calcium influxes and currents due to cell swelling but not to 4αPDD, AA, 5,6-EET, or heat. Furthermore, TRPV4 activation by cell swelling was blocked by CYP epoxygenase inhibitors, further implicating 5,6-EET in mediating this function. The work of Fernandes et al. confirmed the PLA_2_-EET axis in hamster oviductal ciliated cells; however, they also observed that inositol triphosphate from the phospholipase C pathway sensitizes the responses of 5,6-EET to TRPV4.

Finally, the work of Berna-Erro and colleagues^90^ revealed structural determinants for 5,6-EET activity on TRPV4 and a possible binding site. Using molecular docking, they found that 5,6-EET can bind to a pocket in the voltage-sensing-like domains of TRPV4; and although this work precedes the structures of TRPV4 determined experimentally,^91,92^ these later structures show that the proposed 5,6-EET binding site overlaps with the binding site of 4αPDD.^92^ The docking studies of Berna-Erro et al.^90^ revealed that residues K535 and R594 form important ionic interactions with the carboxylic headgroup of 5,6-EET. Mutating K535 *in silico* to an alanine showed reduced binding of 5,6-EET. These data were then confirmed experimentally using a microscale thermophoresis binding assay, where the mutations K535A and R594A showed impaired 5,6-EET binding compared to WT. As mutating R594 is known to reduce TRPV4 activity overall (in the absence of ligand), K535A was taken for further analysis. Calcium influx assays showed that K535A abrogated the responses of TRPV4 to 5,6-EET but not to a synthetic agonist, demonstrating the specificity of this residue toward 5,6-EET and that it does not affect channel activity overall. Finally, they showed that K535A also reduces the response of TRPV4 to osmotic stress using TRPV4-transfected HEK293 whole cell electrophysiology recordings, further supporting the involvement of K535A (and thus 5,6-EET) in TRPV4-mediated mechanosensation.

#### 3.2.2 TRPA1

TRPA1 is best known for detecting noxious nucleophiles, producing a painful, itching sensation to alert our body to the assault. Several exogenous electrophiles (e.g., allicin from garlic, allyl isothiocyanate from mustard and wasabi, and cinnamaldehyde from cinnamon^93^) and endogenous electrophiles (e.g., the prostaglandins PGA2 and PGJ2 and the lipid-breakdown product 4-hydroxynonenal^94,95^) are known activators of this channel. These electrophiles work by covalently binding a cysteine-rich nexus on the cytosolic side of TRPA1, leading to channel opening.^96,97^ Marco Sisignano and colleagues^98^ discovered that 5,6-EET levels increase after nociceptor activation in dorsal root ganglia (DRG) *in vitro* and *in vivo*. There were no significant changes to the levels of the other EET regioisomers. 5,6-EET was able to evoke calcium influx in DRG that was not mediated by 5,6-diHET and which persisted in the presence of COX inhibitors, indicating that the effects of 5,6-EET are not due to further metabolism (see Section 3.3 below). A pharmacological screen using specific TRP channel inhibitors showed that only an antagonist for TRPA1 blocked the effects of 5,6-EET. 5,6-EET had no effects on HEK293 cells expressing TRPA1 with an inactive cysteine-rich nexus, showing that binding to this nexus is important in mediating its function. Finally, it was demonstrated that 5,6-EET elicits neuronal firing and mechanical allodynia via TRPA1 in mouse models. Taken together, these data demonstrate that DRG stimulation (including inflammation) produces 5,6-EET, specifically, which mediates inflammatory pain via TRPA1.

#### 3.2.3 TRPV1

TRPV1 is structurally akin to TRPV4, yet their physiology is distinct. TRPV1 is expressed in primary sensory afferent neurons that are responsible for producing a painful sensation upon activation by heat, chemical agonists, and acid.^99^ It is well-established that TRPV1 is regulated by several inflammatory lipids, including phosphoinositides.^100,101^ Liposome patch recordings and cryogenic electron microscopy (cryo-EM) structural studies demonstrate that phosphoinositides directly bind to a key regulatory site of TRPV1 known as the vanilloid binding pocket (VBP)^102−105^ and that these phosphoinositides keep the channel in a closed conformation.^103,105^ Recently, Arnold et al. demonstrated using cryo-EM that the inflammatory lipid agonist lysophosphatidic acid competes for phosphoinositide binding at this site and helps stabilize an open conformation of TRPV1,^105^ as had been observed for vanilloid agonists.^103,104,106^ Therefore, the direct actions of many other lipid mediators likely involve binding to the VBP.

In another publication,^107^ we demonstrated that epoxide derivatives of the endocannabinoid-like molecules, N-arachidonoyl dopamine (NADA) and N-arachidonoyl serotonin (NA5HT), are TRPV1 ligands. Using a calcium influx assay, we showed that NADA and 14′,15′-epoxy-N-arachidonoyl dopamine (epoNADA) are agonists of TRPV1 (promoting calcium influx) while NA5HT and 14′,15′-epoxy-N-arachidonoyl serotonin (epoNA5HT) act as antagonists (inhibiting capsaicin-evoked influx). Further, epoNA5HT blocks capsaicin-induced calcium influx and currents in native TRPV1(+)-DRG. Importantly, epoNA5HT was shown to be a much more potent antagonist of TRPV1 activity compared to the parent NA5HT compound. It is currently unclear how the epoxidation of NA5HT would increase its efficacy on TRPV1. The structures of lipids bound to TRPV1 thus far do not show any polar residues within the VBP that would interact with the epoxidation site on the tail. Understanding how epoxidation affects lipid binding to TRPV1 would provide key insights into how receptors recognize the epoxide in general.

### 3.3 Commentary on 5,6-EET

As 5,6-EET shows specific interactions with TRPV4 and TRPA1, it is important to discuss this regioisomer in respect to the other regioisomers. The physiological responses of 8,9-EET; 11,12-EET; and 14,15-EET have been the best characterized thus far. It is also these regioisomers that have also shown binding to the putative EET receptor. The role of 5,6-EET in physiology has been controversial due to the difficulties of studying this molecule. One issue is that 5,6-EET can be metabolized by COX enzymes into epoxy-prostaglandins^108−110^ that exert distinct effects compared to EETs.^111^ The stability of 5,6-EET is another issue that obfuscates studies. Among the 4 regioisomers of EETs, 5,6-EET is the least stable in biological media (half-life of about 8 min), owing to the ease of nonenzymatic hydrolysis to the corresponding diol that, in turn, can form a δ-lactone with the carboxyl group.^112^ Therefore, it is quite difficult to determine for sure which species of 5,6-EET is mediating the observed effects: 5,6-EET; 5,6-diHET; or 5,6-diHET-δ-lactone. Furthermore, the instability makes it difficult to accurately quantify the amount of 5,6-EET present in biological tissue, though it is generally found to be lower compared to the other regioisomers, which reflects the regioselectivity of the major CYP epoxygenases.

It is interesting to note that 5,6-EET was the only regioisomer to show appreciable activation of TRPV4 and TRPA1, whereas the other regioisomers bind to anti-inflammatory GPCRs. This shows a functional dichotomy of the EET pathway, in which 5,6-EET can serve as a pro-inflammatory mediator via conversion to 5,6-epoxy-PGH_2_ and binding to TRPA1. In the case of TRPA1, the reactivity and electrophilic nature of 5,6-EET (or the δ-lactone) make this regioisomer a logical agonist. It would be interesting to determine how 5,6-EET may influence inflammation via TRPV4, as vasodilation and cell swelling occur during inflammation^113^ and both of these TRPV4-mediated effects are produced by 5,6-EET. Further studies would have to be done to confirm the inflammatory dichotomy of 5,6-EET.

Given the distinct functions of 5,6-EET, coupled with the fact that Marco Sisignano and colleagues^98^ determined that 5,6-EET was the only regioisomer whose levels increased after DRG stimulation, it stands to reason that there would be an enzyme that specializes in producing 5,6-EET. However, 5,6-EET is often a minor product of known CYP epoxygenases, which produce the other regioisomers in higher quantities. Among the 57 CYP enzymes present in the human genome, 13 remain orphaned, i.e., their primary substrates have yet to be validated.^114^ Therefore, it is perhaps one of these orphaned CYPs that produces 5,6-EET specifically and in great enough quantity to meet these proposed biological functions.

In conclusion, evidence is emerging that the physiology of 5,6-EET is unlike the other regioisomers. With its propensity to bind to pro-inflammatory receptors, either directly or through further metabolism via COX enzymes, this regioisomer is the “black sheep” of EETs. However, more careful and thorough studies are needed to confirm this observation, especially given the chemical instability of this regioisomer. Stable analogues, such as those developed by Campbell and colleagues,^115^ may aid these efforts.

## 4 Discovery of Receptors for Similar Lipid Mediators

Several lipid mediators with structures (and functions) similar to EETs have been discovered. What can we learn about the discovery of these receptors that can help us in finding the putative EET receptor? In this section, we will review recently identified GPCRs that bind similar lipids, which can be used as positive examples for the identification of the EET receptor.

### 4.1 Sphingosine-1-Phosphate Receptor 1 (S1PR1) as a Receptor for 17,18-EEQ

In an effort to understand the atheroprotective effects of EPA, Zhou and colleagues expanded on previous work and identified S1PR1 as a 17,18-EEQ receptor.^116^ S1PR1 is abundantly expressed in endothelial cells and regulates vasodilation, heart rate, and immune cell trafficking.^117^ Using two canonical stimuli for HUVEC activation (oscillatory shear stress and TNF-α), EPA elicited a concentration-dependent inhibition of the endothelial activation marker, vascular adhesion molecule 1 (VCAM1). Inhibiting CYP2J2 attenuated this result, suggesting that an EEQ is responsible for inhibiting activation. They confirmed this conjecture by showing that 17,18-EEQ is 100 times more potent than EPA at inhibiting VCAM1.

Zhou and colleagues proceeded to use RNA sequencing of the HUVECs to identify candidate GPCRs mediating this effect. Of the 80 most highly abundant GPCRs, only 10 enhanced VCAM1 expression; of these 10, only the silencing of S1PR1 prevented 17,18-EEQ-mediated VCAM1 inhibition. From these data, they tested 17,18-EEQ binding to S1PR1 directly. 17,18-EEQ inhibited the binding of radiolabeled sphingosine-1-phosphate (the canonical ligand of S1PR1), thus confirming that 17,18-EEQ binds to S1PR1. Interestingly, a FlAsH-BRET assay showed that 17,18-EEQ induced a different conformational change to S1PR1 compared to sphingosine-1-phosphate. Consistent with this observation, sphingosine-1-phoshate induced G_i_ activation through S1PR1, but 17,18-EEQ biased S1PR1 toward G_q_ and G_11_ signaling. Molecular docking showed that 17,18-EEQ binds to a hydrophobic orthosteric pocket of S1PR1. The docking indicated residues that interact with the carboxylate headgroup and the tip of the hydrophobic tail, which was confirmed with *in vitro* binding assays. However, the authors did not mention or explore residues that specifically interact with the epoxide; therefore, there is no data giving insight into the greater selectivity of 17,18-EEQ over EPA.

Zhou and colleagues further elucidated the pathway by showing that the 17,18-EEQ phenotypes are mediated by endothelial nitrous oxide synthase via TNF-α. They further performed a series of *in vivo* studies investigating the atherosclerotic effect of the EEQ/S1PR1 pathway. Using a mouse model for atherosclerosis, they observed an inhibition of adhesion markers (e.g., VCAM1) in the carotid artery when treated with 17,18-EEQ, which was not observed in S1PR1-knockout mice. Similarly, 17,18-EEQ treatment restored endothelial nitrous oxide synthase phosphorylation. Furthermore, 17,18-EEQ was able to reduce the atherosclerotic lesion area in carotid arteries compared to the S1PR1-knockout. Zhou and colleagues concluded their study by investigating the actions of Vascepa, a stabilized EPA ethyl ether that has recently been approved by the Food and Drug Administration as a prescription drug to treat cardiovascular events. They found that administering Vascepa to mice resulted in an increase in 17,18-EEQ exclusively compared to the other regioiosmers; furthermore, the atheroprotective effects of the drug are abolished in the S1PR1-knockout mice. These data further support that 17,18-EEQ is the primary mediator of EPA-mediated atheroprotection via activation of S1PR1.

### 4.2 Epoxides and Cannabinoid Receptors

The canonical cannabinoid receptors are the GPCRs CB1 and CB2, which were identified from 1988 to 1992 as mediating the effects of phytocannabinoids from cannabis.^118−120^ Soon after, their endogenous ligands, the endocannabinoids AEA and 2-arachidonoyl-glycerol (2-AG) (Figure 2), were discovered in 1992 and 1995, respectively.^121−123^ CB1 is mainly expressed in brain tissue but can also be found in the liver, lungs and kidneys. CB2 is mainly expressed in the immune system and hematopoietic cells. Given the fact that endocannabinoids are PUFAs with functionalized headgroups, it is unsurprising that endocannabinoids are efficiently metabolized by epoxygenases, producing epoxy-endocannabinoids that have distinct functions. Using competition binding assays, Snider and colleagues^124^ showed 5,6-EET-EA is a potent and selective CB2 receptor agonist in Chinese hamster ovary (CHO) cells expressing either recombinant human CB1 or CB2. 5,6-EET-EA showed greater than 300-fold affinity for the CB2 as compared to CB1 receptor, and more than 1000-fold affinity for CB2 compared to AEA. Previously, activation of CB2 was shown to reduce cAMP levels within cells via its associated G-proteins G_i/o_.^125^ In CHO cells, treatment of CB2-CHO cells with 5,6-EET-EA decreased the intracellular cAMP levels despite pretreatment with forskolin (adenylate cyclase activator). Synthetic CB2 agonist (AM1241) also reduced intracellular cAMP levels in forskolin pretreated cells. These results confirm the functional activation of the CB2 receptor in 5,6-EET-EA-induced CHO cells. Furthermore, there was an increased synthesis of 5,6-EET-EA in microglia stimulated by interferon γ (IFN-γ).^124^ Significant upregulation of CB2 had previously been shown in response to IFN-γ-treatment in microglia cells.^126^ 5,6-EET-EA may thus signal via CB2 to perform its inflammatory role in these cells.

Two significant epoxides of 2-AG are 2–11,12-EG and 2–14,15-EG. In radioligand binding experiments,^127^ both regisomers of 2-EG were shown to bind to CB1 and CB2 receptors similarly to 2-AG. High-affinity binding of 2–11,12-EG was further confirmed in transfected CHO cells expressing either human CB1 or CB2. In these cells, 2–11,12-EG induced activation of downstream p44/p42 ERKs (extracellular signal-regulated kinases), which could be inhibited by CB1 or CB2 antagonism and pertussis toxin, but not by adenylate cyclase, protein kinase A, or phospholipase C inhibitors, which suggested the involvement of receptors coupled to G_i/o_ proteins. 2-EG also activated ERK signaling in N182G2 neuroblastoma cells, which express endogenous CB1 but not CB2 receptors. This effect was inhibited by CB1 antagonism and pertussis toxin, again pointing to ERK signaling in these cells via CB1 receptors coupled to G_i/o_ proteins. Similarly, selective CB2 antagonism and pertussis toxin blocked 2-EG-induced ERK signaling in human promyelocytic leukemia HL-60 cells, which express functional CB2 but not CB1 receptors. Furthermore, 2-EG-induced hypomotility and hypothermia responses in intact mice, and vasorelaxation of renal glomerular afferent arterioles were linked to CB1. 2-EG also induced migration in HL-60 cells via CB2 signaling. Thus, the authors show that 2-EG signals via both CB1 and CB2, activating ERKs.

We have shown that ω-3 endocannabinoid epoxides bind to CB2 to carry out their anti-inflammatory functions.^42^ Like AA, EPA and docosahexaenoic acid (DHA) can be conjugated to an ethanolamide to produce EPEA and DHEA, respectively. These can then be epoxidized to form several regioisomers of EEQ-EAs and EDP-EAs, respectively. Working with microglial cells, we showed that both 17,18-EEQ-EA and 19,20-EDP-EA inhibited the production of pro-inflammatory biomarkers, IL-6 and NO, with increased production of the anti-inflammatory cytokine IL-10. The anti-inflammatory effects of these ω-3 endocannabinoid epoxides were partially blocked by a CB2 antagonist, AM630. CB2 ligands have been shown to promote the anti-inflammatory macrophage phenotype.^128,129^ In our work, a PRESTO-Tango β-arrestin recruitment assay demonstrated preferential binding of EPEA and DHEA epoxides to CB2 as compared to CB1 and identified 19,20- and 16,17-EDP-EA as potent CB2 agonists. A comparison of the anti-inflammatory properties of EDP-EA and EEQ-EA with their respective parent compounds showed that EDP and EEQ epoxides have improved anti-inflammatory properties. Similarly, EPEA and DHEA epoxides were more potent functional activators of CB2 as compared to their parent compounds, so their increased anti-inflammatory properties can be linked to CB2 activation.

Later, we investigated the antitumorigenicity of ω-3 endocannabinoid epoxides in model metastatic and nonmetastatic cell lines.^45^ In mouse lungs that developed tumors from tail-vein injections of K7M2 osteosarcoma cells, levels of DHEA epoxides were upregulated in comparison to noninfected lungs. The effects of these epoxides on cell viability, apoptosis, and tissue migration were tested and we found that 10,11-EDP-EA was the most effective in reducing cell viability and tissue migration while promoting apoptosis in HOS, 143B, and MG63 cell lines. Tissue migration (determined by a scratch assay) was shown to be partly mediated by CB1, as the coadministration of the CB1 antagonist rimonabant partially ameliorated these effects. Derivatives of 10,11-EDP-EA were then developed to increase the potency while limiting the susceptibility to degradation by fatty acid amide hydrolase and sEH. 10,11-EDP was conjugated to isopropyl, cyclopropyl, and n-propyl amide derivatives. 10,11-EDP-EA and these derivatives all showed greater binding to CB1 than CB2 in PRESTO-Tango assays. However, the pro-apoptotic effect of the EDP-EA epoxides was not significantly affected when CB1 or CB2 antagonists were coadministered, demonstrating that the pro-apoptotic effects are not significantly mediated by these cannabinoid receptors. Therefore, there may be further unknown receptors of these endocannabinoid epoxides: either putative unknown cannabinoid receptors or potentially the unknown EET receptor.

### 4.3 Identification and Characterization of Resolvin D2 Receptor

D-Series resolvins (RvDs) are DHA-derived resolution phase mediators and E-series resolvins (RvEs) are EPA-derived mediators. Together, with other potent endogenous mediators, they are known as specialized pro-resolving mediators, or SPMs. SPMs have been shown to interact with cell-surface GPCRs on leukocytes to limit polymorphonuclear neutrophil (PMN) infiltration and stimulate phagocyte resolution.^130^ RvD1 directly activates GPR32 (now designated DRV1), whereas RvE1 activates two separate receptors, ChemR23/ERV1 and BLT1.

The identification of an RvD2 receptor emerged from an unbiased GPCR-arrestin–coupled custom commercial screening system.^131^ Among 77 orphan human GPCRs analyzed, three receptors (GPR18, GPR26, and GPR30) gave strongest chemiluminescence signals in response to 10 nM of RvD2. RvD2 did not activate GPR32, a human receptor for RvD1 or GPR31, a recently reported receptor for 12S-hydroxy-eicosatetraenoic acid. Further analysis of GPR18, GPR26, and GPR30 using a β-arrestin–based ligand receptor interaction system showed increased chemiluminescence signal only in cells overexpressing GPR18. RvD3 at similar concentrations (10^–14^–10^–8^ M) did not activate GPR18, suggesting selective activation of GPR18 by RvD2. Since leukocytes are a known target for RvD2, GPR18 expression on human leukocytes was confirmed in peripheral blood PMN, peripheral blood monocytes, and monocyte-differentiated macrophages using flow cytometry. RvD2 dose-dependently (1–100 nM) elicited rapid changes in impedance in CHO cells overexpressing recombinant human GPR18. Changes in impedance were significantly reduced when cells were incubated with anti-hGPR18 antibody before addition of RvD2. Cholera toxin prominently inhibited RvD2-initiated impedance changes suggesting that RvD2 triggered GPR18 coupling to G_S_ in CHO-hGPR18 cells. As RvD2 activation of GPR18 in CHO cells was likely mediated via a G_S_-like protein, it was further investigated whether RvD2 regulates cAMP in human macrophages. RvD2 was found to significantly increase cAMP with human macrophages, and this action was weakened when macrophages were transfected with shRNA targeting GPR18. Finally, radiolabeled ligand binding was utilized to confirm high affinity and specific binding of RvD2 with recombinant human GPR18. Tritium labeled RvD2 methyl ester was used for saturation binding with recombinant hGPR18 expressed in CHO cells in the absence or presence of 10 μM unlabeled RvD2-methyl ester. Specific binding was obtained and Scatchard plot analysis produced a *K*_d_ of 9.6 ± 0.9 nM which is within the bioactive range of RvD2. RvD2 and RvD2-methyl ester gave similar affinities to CHO-GPR18 with IC50–100 nM. SPMs like RvD1, RvD3, maresin 1 (MaR1), and protectin D1 (PD1) did not significantly compete for [^3^H]-RvD2-ME–specific binding.

To confirm the GPR18 mediated RvD2s pro-resolving actions, several *in vitro* and *in vivo* experiments were performed. Overexpression of hGPR18 in human macrophages significantly increased phagocytosis of fluorescently labeled STZ (serum-treated zymosan), *E. coli*, and apoptotic PMN, while knockdown of endogenous macrophage GPR18 using shRNA significantly abolished these RvD2-stimulated actions. Overexpression of GPR18 dose-dependently (0.1–10 nM RvD2) increased the expression of CD206 and CD163. CD206 and CD163 are phagocytic receptors and markers of the anti-inflammatory and pro-resolving phenotype, thus indicating that phagocytic receptors CD163 and CD206 contribute to RvD2-enhanced macrophage phagocytosis. In an acute murine peritonitis model, RvD2 limited PMN infiltration and enhanced efferocytosis in a GPR18-dependent manner, while GPR18-deficient mice displayed impaired resolution of infections and diminished responses with RvD2. In a hind limb I/R (ischemia–reperfusion) model of second organ lung injury, no statistically significant difference was found in PMN numbers between WT and GPR18-KO mice. Similarly, in *S. aureus* skin infection, the ability to clear bacteria or PMN infiltration was not compromised in GPR18-KO mice 4 h after initiation of infection. Thus, it is quite possible that in sterile lung injury and in the acute phase of infection, endogenous RvD2 is not produced and/or does not play an essential protective role. In both these cases, exogenous administration of RvD2 in WT mice rendered marked protection, which was lost in GPR18-KO mice.^131^

### 4.4 Identification and Functional Analysis of GPR75, a Specific Receptor for 20-HETE

20-Hydroxyeicosatetraenoic acid (20-HETE) is one of the principle CYP eicosanoids and its increased levels are associated with hypertension, stroke, myocardial infarction and vascular diseases.^132^ The presence of a receptor/binding site for 20-HETE has been suggested by the development of specific analogs and antagonists that mimic or block many of its known biological functions. In a study by Schwartzman and colleagues,^133^ using cross-linking analogs, click chemistry, binding assays, and functional assays, GPR75 was identified as a specific high-affinity receptor for 20-HETE. They first synthesized a 20-HETE analog, 20-APheDa, which contained a benzophenone (photoreactive cross-linker) and an azide for selective binding and labeling to a click-chemistry reagent (dibenzocyclooctyne (DBCO) 800CW Infrared Dye). Incubation of 20-APheDa with membrane fractions of human ECs (20 μg) followed by UV cross-linking and incubation with the click reagent yielded several bands, including a dominant band located at 47–49 kDa. Identification of the dominant band was carried out by peptide fingerprint mass mapping (using MS data) and peptide fragmentation mapping (using MS/MS data). Sequence analysis identified several proteins including HIC-5 (peroxide-inducible clone 5) and domains and proteins associated GIT1 (G protein coupled receptor kinase interactor 1). This information was used for protein partner analysis which revealed a candidate orphan receptor GPR75.

In competition binding assay, 20-HETE but not 12(S)-HETE displaced bound [^3^H8] 20-HETE (6.67 nM) from EC membranes in a concentration-dependent manner with a calculated K_d_ (3.75 nM) well within the concentration range of 20-HETE biological actions. Knockdown of GPR75 prevented ^3^H-20-HETE binding to EC membranes. Likewise, addition of the 20-HETE antagonist inhibited ^3^H-20-HETE binding to EC membranes. Further experiments showed that 20-HETE increases IP-1 accumulation in EC but not in GPR75-deficient cells. 20-HETE also rapidly dissociated G_q/11_ from GPR75. Using immunoprecipitation and immunoblotting experiments, GPR75 was shown to be expressed in the vascular endothelium and associated with G_q/11_, GIT1, and HIC-5. Further, 20-HETE was shown to alter the association of GPR75 with Gq/11, GIT1, and HIC-5. All together, these results indicated that 20-HETE binds to EC membranes via, pairs with GPR75 and functionally activates it, which is coupled to G_q/11_.^133^

## 5 Conclusion

There are numerous data supporting the existence of a high-affinity GPCR mediating the effects of EETs. Despite the therapeutic potential of the EET pathway in remediating cardiovascular and inflammatory diseases, the identity of this receptor has remained elusive. The identification of other lipid receptors provides hope that the putative EET receptor may be found, and receptors like S1PR1 can serve as a model for the discovery of the putative EET receptor. There are several key points about this study that highlight some of the nuances. 1) A successful approach may involve using primary cells or cells that naturally express the putative EET receptor. 2) The receptor may have already been discovered and has a different canonical activator. 3) The EETs could be biasing the receptor to be G_S_-coupled when it normally is not. Our understanding of GPCRs and our biological toolbox have advanced greatly since the search began for the receptor, providing hope that we may be successful with a revitalized search. It is worth reanalyzing previous attempts with these new advances in mind.

Additionally, the success may lie in chemistry. Many receptors are discovered by hunting down the receptors for drugs. For example, both the cannabinoid receptors and TRPV1 were determined to be the receptors for tetrahydrocannabinol and capsaicin, respectively, before it was found that AEA binds to these receptors. Chemical analogues and drugs may be one way in which we can discover the EET receptor. The stability of the epoxide, a fundamentally reactive moiety, may frustrate efforts compared to other lipids. Currently, there is only one effective, commercially available EET receptor antagonist, and having other, more stable antagonists available, may help more researchers in the search. Several studies producing EET-like inhibitors demonstrate the feasibility of adding more inhibitors to the commercial milieu.^134,135^ One of the key issues is that the epoxide itself appears vital to the receptor’s recognition of EETs, as chemical analogues of epoxides such as thiirane often fail to elicit a response when the putative receptor is directly probed. Although several epoxide analogues have been developed that show EET-like activity, these studies have not directly probed the putative receptor in their function. Furthermore, developing analogues of lipids in general is a challenge, which hampers interest in the medicinal chemistry community.

The search for the EET receptor is also confounded by the “dirty” nature of these molecules. Several diverse receptors have already been discovered that directly bind to and mediate EET physiology, which provides for a lot of “off targets” and “distractions” when searching for the putative receptor. But maybe this ubiquity is their strength; perhaps it is the concerted efforts of many proteins that give rise to their potent phenotypes. Perhaps we are not looking for one particular receptor, and several GPCRs comprise the 47-KDa band discovered by Chen and colleagues.^64^ However, we will never know unless we continue to search for the White Whale of the EET world.
